# Effect of SDS on release of intracellular pneumocandin B_0_ in extractive batch fermentation of *Glarea lozoyensis*

**DOI:** 10.1007/s00253-019-09920-x

**Published:** 2019-06-03

**Authors:** Kai Yuan, Baoqi Huang, Tingting Qin, Ping Song, Ke Zhang, Xiaojun Ji, Lujing Ren, Sen Zhang, He Huang

**Affiliations:** 1 College of Biotechnology and Pharmaceutical Engineering, Nanjing Tech University, 30 South Puzhu Road, Nanjing, 211816 China; 2 School of Food Science and Pharmaceutical Engineering, Nanjing Normal University, 1 Wenyuan Road, Nanjing, 210023 China; 3 Jiangsu Collaboration Innovation Center of Chinese Medical Resources Industrialization, College of Pharmacy, Nanjing University of Chinese Medicine, 138 Xianlin Road, Nanjing, 210023 China

**Keywords:** Pneumocandin B_0_, Extractive batch fermentation, Extractant, Membrane permeability, Morphology

## Abstract

Pneumocandin B_0_ is a hydrophobic secondary metabolite that accumulates in the mycelia of *Glarea lozoyensis* and inhibits fungal 1,3-β-glucan synthase. Extractive batch fermentation can promote the release of intracellular secondary metabolites into the fermentation broth and is often used in industry. The addition of extractants has been proven as an effective method to attain higher accumulation of hydrophobic secondary metabolites and circumvent troublesome solvent extraction. Various extractants exerted significant but different influences on the biomass and pneumocandin B_0_ yields. The maximum pneumocandin B_0_ yield (2528.67 mg/L) and highest extracellular pneumocandin B_0_ yield (580.33 mg/L) were achieved when 1.0 g/L SDS was added on the 13th day of extractive batch fermentation, corresponding to significant increases of 37.63 and 154% compared with the conventional batch fermentation, respectively. The mechanism behind this phenomenon is partly attributed to the release of intracellular pneumocandin B_0_ into the fermentation broth and the enhanced biosynthesis of pneumocandin B_0_ in the mycelia.

## Introduction

Pneumocandin B_0_, an antifungal agent produced by *G. lozoyensis*, is a lipohexapeptide of the echinocandin family that inhibits fungal 1, 3-β-glucan synthase (Chen et al. 2016). In 1987, pneumocandin B_0_ was discovered among various minor components of pneumocandin fermentations. This minor component was chosen as natural starting material for the synthesis of caspofungin acetate (CANCIDAS®) (Balkovec et al. 2014; Schwartz et al. 1989). *G. lozoyensis* was recognized as a novel fungus through DNA fingerprinting and rDNA sequence analysis (Bills et al. 1999). Because of the difficulty in used in traditional protoplast transformation techniques with this fungus, *Agrobacterium*-mediated transformation was developed as a simple and efficient in gene replacement method (Zhang et al. 2003). Pneumocandin biosynthetic gene clusters have been characterized, providing a blueprint for engineering new pneumocandin derivatives with improved pharmacological properties (Chen et al. 2013). In addition, some strategies, such as strain mutagenesis (Masurekar et al. 1992), amino acid and trace element supplementation (Petersen et al. 2001), and osmotic stress control strategy (Song et al. 2018), were applied in fermentation processes to improve the pneumocandin B_0_ yield. Echinocandins, including pneumocandin B_0_, are hydrophobic secondary metabolites that accumulate in the mycelia (Bills et al. 2015). Many intracellular products are not easily released into the fermentation broth and can result in product feedback inhibition (Wang et al. 2013). To alleviate intracellular accumulation of metabolites, the strategy of enhancing the release of products outside the cell membrane by weakening the cells’ permeability barrier has been suggested (Liang et al. 2010).

Extractive fermentation technology has been successfully applied as an effective method for improving the extraction of fungal intracellular products (Kleinegris et al. 2011; Wang and Dai 2010). With the addition of extractive agents in the fermentation broth, the micellare aqueous solution can separate into two phases, where one is a dilute phase (aqueous solution) while the other is a coacervate phase (extractant-rich phase). Intracellular products are released from the intracellular to the extracellular and progressively extracted into the coacervate phase. Thus the product is continuously extracted into the nonaqueous solvent phase and the fungal cells continuously produce hydrophobic metabolites (Chen et al. 2017a; Hu et al. 2012). Compared with the traditional submerged cultivation, the extractive fermentation exhibits some important advantages, such as the higher accumulation of hydrophobic secondary metabolites and reduced effort in downstream solvent extraction (Anvari et al. 2009; Oda et al. 2015). For example, with the addition of EDTA to *Pseudomonas putida* G7 fermentations, the Ca^2+^ and Mg^2+^, stabilizing the outer membrane structure by bonding the lipopolysaccharides to each other, were removed and the lipopolysaccharides detached, resulting in perforations in areas of the outer walls and reduction of 76.71% of limonene in citrus juice (Malik et al. 2012). Triton X-100 was used for the extraction of substrates into the micelle pseudophase and increased substrate biosynthesis in fermentations of *Pseudomonas putida* and *S. cerevisiae* (Chen et al. 2017b; Xue et al. 2010).

In this work, we investigated the influence of different extractants on the pneumocandin B_0_ yield and explored the possible reason for improved pneumocandin B_0_ production in the extraction system. Moreover, the response of the putative trans-membrane secretion model of pneumocandin B_0_ in extractive fermentation was established accordingly.

## Materials and methods

### Microorganism and fermentation medium

*Glarea lozoyensis* CCTCC M 2019020 Q1 is preserved in the China Center for Type Culture Collection and it is a mutant of *Glarea lozoyensis* ATCC 74030.

The seed medium was composed of glucose 40 g/L, soybean powder 20 g/L, KH_2_PO_4_ 1 g/L, and trace element solution 10 mL, and the initial pH was adjusted to 5.0. The trace element solution was described in a previous study (Qin et al. 2016).

The fermentation medium was composed of glucose 20.0 g/L, D-mannitol 80 g/L, soybean meal 20 g/L, and K_2_HPO_4_ 2.5 g/L, and the initial pH was 6.8.

### Batch fermentation and extractive batch fermentation

For the shake-flask batch fermentation in Erlenmeyer flasks, mycelia growing on potato dextrose agar (PDA) slants were transferred to 250-mL Erlenmeyer flasks containing 50 mL seed medium and cultivated at 25 °C and 220 rpm for 5 days. Then, 10% (*v*/*v*) of the preculture was used to inoculate in 50 mL of fermentation medium, which was cultured at 25 °C and 220 rpm for 17 days. Extractive batch fermentation was performed in the same manner as the batch fermentation except that different extractants were added into 50 mL of fermentation medium at different times.

### Measurement of dry cell weight, pneumocandin B_0_, and SDS

The measurement of dry cell weight (DCW) and the analytical methods used to determine the concentrations of pneumocandin B_0_ were described in our previous work (Qin et al. 2016). The pneumocandin B_0_ yield in the supernatant and mycelium was processed by the following method: 1 mL of the fermentation broth was centrifuged at 3000×*g* for 10 min and the supernatant was collected. The supernatant was diluted 5 times with ethanol and then measured by HPLC. The yield of pneumocandin B_0_ in the mycelium was equal to the yield of pneumocandin B_0_ in the fermentation broth minus the yield of pneumocandin B_0_ in the supernatant.

SDS was detected by gas chromatography. 1 mL of the fermentation broth was centrifuged at 3000×*g* for 10 min, and the supernatant was collected and sulfuric acid was used to hydrolyze SDS to 1-dodecanol (Liu et al. 2009). The analysis was separated on an HP-5 column (30 m × 0.53 mm, 1.5 μm; Agilent Technologies Inc., USA) with nitrogen as the carrier gas and was detected using an FID detector. The temperature program was as follows: starting temperature is 80 °C. One minute later, temperature rises to 260 °C, with rising rate 10 °C/min. Then, the samples were loaded directly, and the concentration of 1-dodecanol was determined by peak area normalization and SDS was determined by external standard method in which 1-dodecanol was used as the reference substance.

### Assay for the physiological performance of cell membranes and GC analysis of the cellular fatty acid composition

The fatty acid composition was analyzed according to the method described by Wang et al. (2013) with some modifications. About 35 mL of fermentation broth was centrifuged at 8000×*g* for 10 min at 4 °C, washed twice with distilled water, and resuspended in the original volume of distilled water in 50 mL tubes. Then, cell disruption was conducted using a JY 92-IIN ultrasonicator (Scientz Bio, China). Afterwards, 70 mL of freshly prepared extraction reagent (ethyl alcohol/n-hexane, 1:1 *v*/*v*) was added to each tube and the tubes were shaken. After centrifugation at 5000×*g* for 2 min, the lighter (n-hexane) phase was collected and evaporated to dryness by rotary evaporation at 65 °C. The obtained lipids were resuspended in 3 mL of saponification solution (0.5 mol/L KOH in methanol). Lipid saponification was performed in a water bath at 65 °C for 17 min, after which the mixture was cooled to room temperature. Then, 2 mL of methylation solution (BF3-diethyl etherate/methanol, 3:7 *v*/*v*) was added to the mixture and methylation was performed in a water bath at 65 °C for 7 min. After cooling to room temperature, 2 mL of saturated NaCl solution and 3 mL of n-hexane were added. After shaking and centrifugation, the upper (n-hexane) phase was collected in a GC vial. The GC method was described in previous paper (Sun et al. 2016).

### Determination of outer membrane permeability

Outer membrane (OM) permeability was determined using the 1-N-phenylnaphthylamine (NPN) assay (Loh and Hancock 1984; Xing et al. 2009) with some modifications. The batch fermentation broth cultivated for 13 days was withdrawn and centrifuged at 4000×*g* for 10 min. The collected mycelia were washed with sterile water three times and resuspended in 0.5% NaCl solution. Then, SDS solution (2.0 g/L) was mixed with 1.5 mL suspension and 20 μL 1 mM NPN, so that the final SDS final concentrations were 0 and 1.0 g/L. Afterwards, the fluorescence was detected by using a Spectra Max M3 spectrophotometer (Molecular Devices, USA) with an excitation wavelength of 350 nm and an emission wavelength of 420 nm.

### Determination of mitochondrial activity

The rhodamine123 (Rh123) assay (Darzynkiewicz et al. 1981) was taken and used to determine mitochondrial activity, with some modifications.

The batch fermentation broth cultivated for 13 days was withdrawn and centrifuged at 4000×*g* for 10 min. The collected mycelia were washed with sterile water three times and resuspended in 0.1 M phosphate buffer. Then, SDS solution (2.0 g/L) was mixed with 1.5 mL suspension at 25 °C for 30 min, keeping the final SDS concentrations at 0.0 and 1.0 g/L. Subsequently, 10 μL of 1 g/L Rh123 was added and the fluorescence was detected by a Spectra Max M3 spectrophotometer (Molecular Devices, USA) with an excitation wavelength of 488 nm and an emission wavelength of 530 nm.

### Morphology analysis of mycelia from batch fermentation and extractive batch fermentation

The morphology of mycelia was investigated using the method described by Chen et al. (2017a, b), with some modifications as follows: 6 mL of batch fermentation broth and extractive batch fermentation broth were withdrawn from the cultivation at the 15th day and washed three times with distilled water via centrifugation at 3000×*g* for 10 min. The collected mycelia then were fixed with 4 mL of 5% glutaraldehyde at 4 °C for 4 h. Then, 4 mL of the mixture was used to measure the diameter of hyphae and pellets. The remaining mixture was collected and washed three times with 0.1 M phosphate buffer via centrifugation at 3000×*g* for 10 min. Then, the samples were successively dehydrated with 30, 50, 70, 85, 95, and 100% (*v*/*v*) ethanol. Subsequently, the solvent was evaporated in an FD-1A-50 freeze-dryer (Zhengqiao, China) for 12 h, and the samples were observed using an SU8010 scanning electron microscope (SEM) (Hitachi, Japan).

### Statistical analysis

The data of the fermentation were presented as the averages of three parallel samples, and the error or error bars indicate the standard deviation from the means of triplicates.

## Results

### Effects of different surfactants on the pneumocandin B_0_ yield and biomass of *G. lozoyensis*

**Fig. 1:**
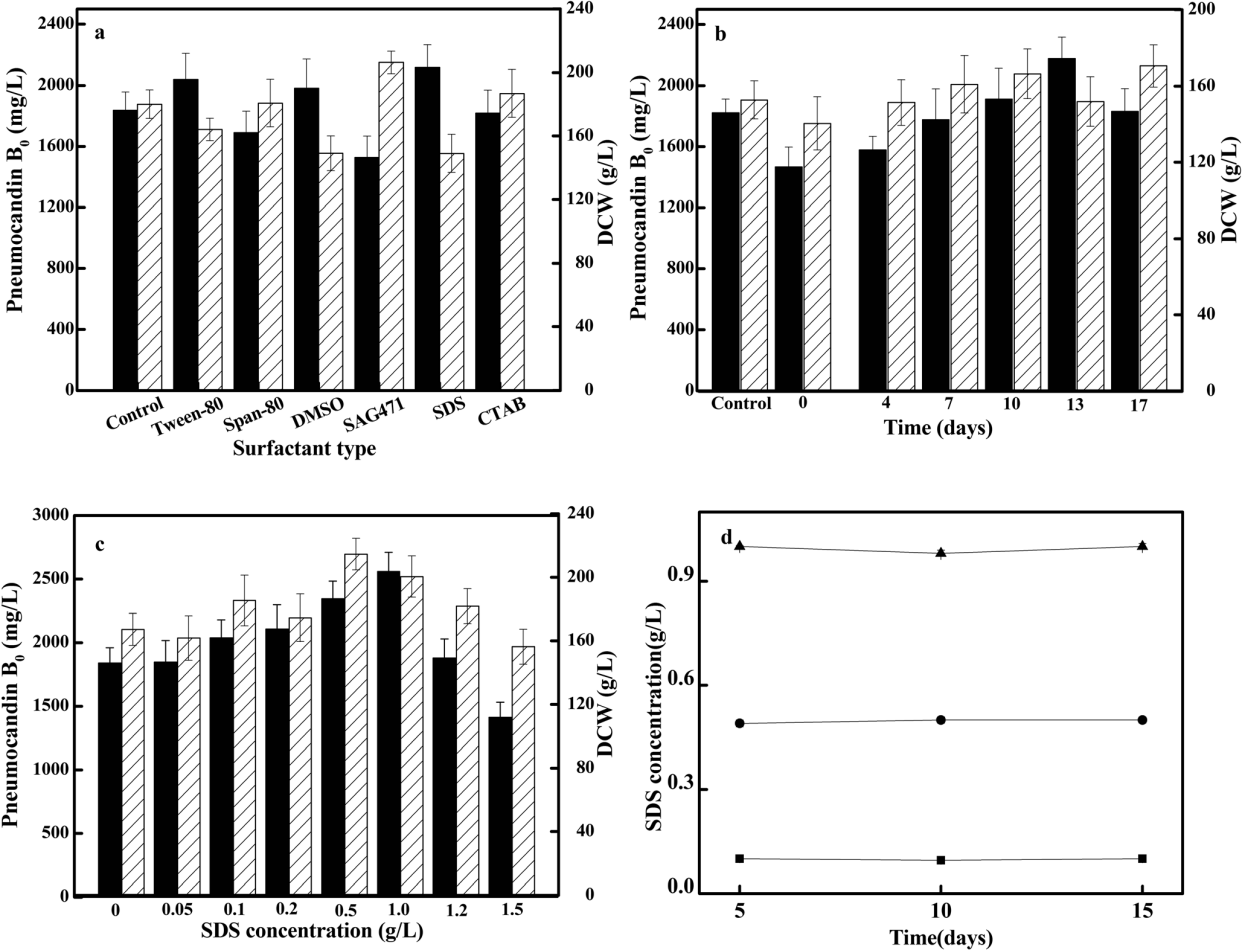
**a** Effects of different extractants on the pneumocandin B_0_ yield and biomass of *G. lozoyensis*; **b** effects of the timing of SDS addition on cell growth and pneumocandin B_0_ yield; **c** effects of the added SDS concentration on cell growth and pneumocandin B_0_ yield (black and striped bars show the pneumocandin B_0_ yield and DCW, respectively; the fermentation finished on day 17); **d** time-profile of SDS concentrations in extractive batch fermentations (black square, 0.1 g/L; black circle, 0.5 g/L; black triangle, 1.0 g/L)

Firstly, the effects of different extractants on *G. lozoyensis* fermentation were investigated. The span-80, tween-80, dimethyl sulfoxide (DMSO), silicone defoaming agent (SAG471), sodium dodecyl sulfate (SDS), and cetyltrimethylammonium bromide (CTAB) were added at 0.1 g/L on day 10 of *G. lozoyensis* fermentation (Fig. 1a). Among the six different extractants, the addition of Tween-80, DMSO, and SDS had a beneficial effect on the pneumocandin B_0_ yield, while the addition of span-80, SAG471, and CTAB promoted the growth of *G. lozoyensis*. Compared to the control group, the pneumocandin B_0_ yield with the addition of SDS reached a maximum of 2085.95 mg/L with 16.2% increase, while the DCW reached 148.99 g/L with 6.42% decrease. Considering the fact that SDS is a pharmaceutical additive and therefore safe in low concentrations, SDS was chosen as the extractant for further experiments.

Secondly, we optimized the addition time of SDS. 0.1 g/L SDS was added at days 0, 4, 7, 10, 13, and 16 of the fermentation (Fig. 1b). When SDS was added at days 0, 4, 7, 10, or 13, the final pneumocandin B_0_ yield increased gradually and reached its maximum. However, with SDS addition at day 17, the pneumocandin B_0_ yield was higher than the control group but not higher than in the previous group.

Thirdly, we optimized the addition concentration under the optimal addition time (Fig. 1c) (Xiong et al. 2015). The production of pneumocandin B_0_ varied significantly depending on surfactant concentrations. When the SDS concentration was less than or equal to 1.0 g/L, it improved the pneumocandin B_0_ yield. With increasing concentration of SDS, the pneumocandin B_0_ yield increased and reached its maximum at a SDS concentration of 1.0 g/L. However, at higher concentrations, the yield of pneumocandin B_0_ decreased sharply. These results indicated that 1.0 g/L SDS was most effective, especially when added on the 13th day of fermentation.

The addition of SDS to the fermentation broth changes the ionic strength, which may cause a change of the osmotic pressure (Yang et al. 2015; Yang et al. 2014). Therefore, whether the change of Na^+^ concentration caused the change of pneumocandin B_0_ yield was further investigated. In contrast to the addition of 1.0 g/L (3.67 mmol/L) SDS, the results showed that the addition of 2.0~5.0 mmol/L NaCl at day 13 did not affect on the accumulation of pneumocandin B_0_, and the low salt concentration did not affect the fermentation process (date not shown). Moreover, at pHs outside a range of approximately 5 to 8, pneumocandin B_0_ undergoes accelerated ionization or ring opening at the hemiaminal (Bouffard et al. 1995). We measured the pH at day 13 with SDS addition. The pH of the control group was about 7.2 and the pH of the test group was about 7.4, which was within a reasonable range and would not affect the structure of pneumocandin B_0_. Taoka et al. (2011) reported that Tween-80 can promote the growth of *Thraustochytrium aureum* as a carbon source. Therefore, the total SDS content in the fermentation during the fermentation process (0~15 days) was examined. The results showed that there was no significant change in the SDS concentration during days 0~15, proving that SDS was not used as a carbon source (Fig. 1d).

### Effects of SDS on the distribution of pneumocandin B_0_ between mycelium

Table 1 compares the normal batch fermentation (control group, 0.0 g/L SDS concentration) and the extractive batch fermentation (test group, 1.0 g/L SDS concentration). Compared to the normal batch fermentation, the pneumocandin B_0_ yield increased 37.63% when the extractive fermentation was finished. In the batch fermentation, the extracellular and intracellular pneumocandin B_0_ yields were 228.67 and 1608.33 mg/L, respectively. The P_2, control group_ % was 12.44 and P_3, control group_ % was 87.56. In the test group, the extracellular and intracellular pneumocandin B_0_ yields were 580.33 and 1947.67 mg/L, respectively. The P_2, test group_ % was 24.29 and P_3, test group_ % was 75.71. ρ_1, control group_ was 9.61 mg/g and ρ_1, test group_ was 9.72 mg/g. ρ_2, control group_ was 10.98 mg/g and ρ_2, test group_ was 12.62 mg/g.

**Table 1:** Effects of adding SDS addition on the intracellular and extracellular pneumocandin B_0_ content and diameters of hyphae and pellet during *G. lozoyensis* fermentation

SDS concentration	Fermentation broth		Supernatant		Mycelium	
	PB_0_ mg/L	P_1_ %	PB_0_ mg/L	P_2_ %	PB_0_ mg/L	P_3_ %
0.0 g/L	1837.33 ± 50.41	100	228.67 ± 10.22	12.44	1608.33 ± 30.24	87.56
1.0 g/L	2528.67 ± 26.22	100	580.33 ± 15.36	24.29	1947.67 ± 29.65	75.71
	Hyphal diameter μm		Pellet diameter nm	DCW g/L	ρ_1_ mg/g	ρ_2_ mg/g
0.0 g/L	0.8563 ± 0.034		0.5261 ± 0.019	167.28 ± 11.72	9.61	10.98
1.0 g/L	0.7386 ± 0.041		0.4915 ± 0.026	200.40 ± 15.65	9.72	12.62

*P*_*1*_, total pneumocandin B_0_ yield in the fermentation broth (defined as 100 %); *P*_*2*_, percentage of the total pneumocandin B_0_ found in the supernatant; *P*_*3*_, percentage of total pneumocandin B_0_ found in the mycelium; *ρ*_*1*_, pneumocandin B_0_ yield in mycelium per DCW; *ρ*_*2*_, pneumocandin B_0_ yield per DCW; the data of the hyphal and pellet diameters were presented as the averages of thirty parallel samples, and the errors indicate the standard deviation

### Effects of SDS on cell membrane composition and mitochondrial activity during extractive fermentation

As shown in Table 2, with the addition of SDS, the content of palmitate (C16:0) and stearate (C18:0) were reduced while that of octadecenoic acid (C18:1), octadecadienoic acid (C18:2), and hexadecatrienoic acid (C18:3n3) increased. When 1.0 g/L SDS was added, the content of C18:1 and C18:2 were 2.3 and 1.7 times higher than in the control group. Thus, the unsaturated/saturated fatty acid ratio and the index of unsaturated fatty acids increased significantly with the addition of the surfactant.

**Table 2:** The effect of SDS on the fatty acid composition of *G. lozoyensis*

SDS (g/L)	C14:0	C15:0	C16:0	C16:1	C17:0	C17:1
0.0	4.23 ± 0.11	4.16 ± 0.15	17.49 ± 0.22	11.92 ± 0.14	7.75 ± 0.13	3.22 ± 0.08
1.0	4.86 ± 0.09	2.68 ± 0.07	9.63 ± 0.21	8.22 ± 0.15	6.21 ± 0.13	3.05 ± 0.11
SDS (g/L)	C18:0	C18:1cis	C18:2	C18:3n3	C20:1n9	C22:0
0.0	11.36 ± 0.20	8.63 ± 0.04	14.37 ± 0.19	2.18 ± 0.07	12.76 ± 0.18	2.00 ± 0.07
1.0	5.11 ± 0.12	20.16 ± 0.25	24.69 ± 0.14	3.77 ± 0.10	9.73 ± 0.23	1.89 ± 0.04

**Fig. 2:**
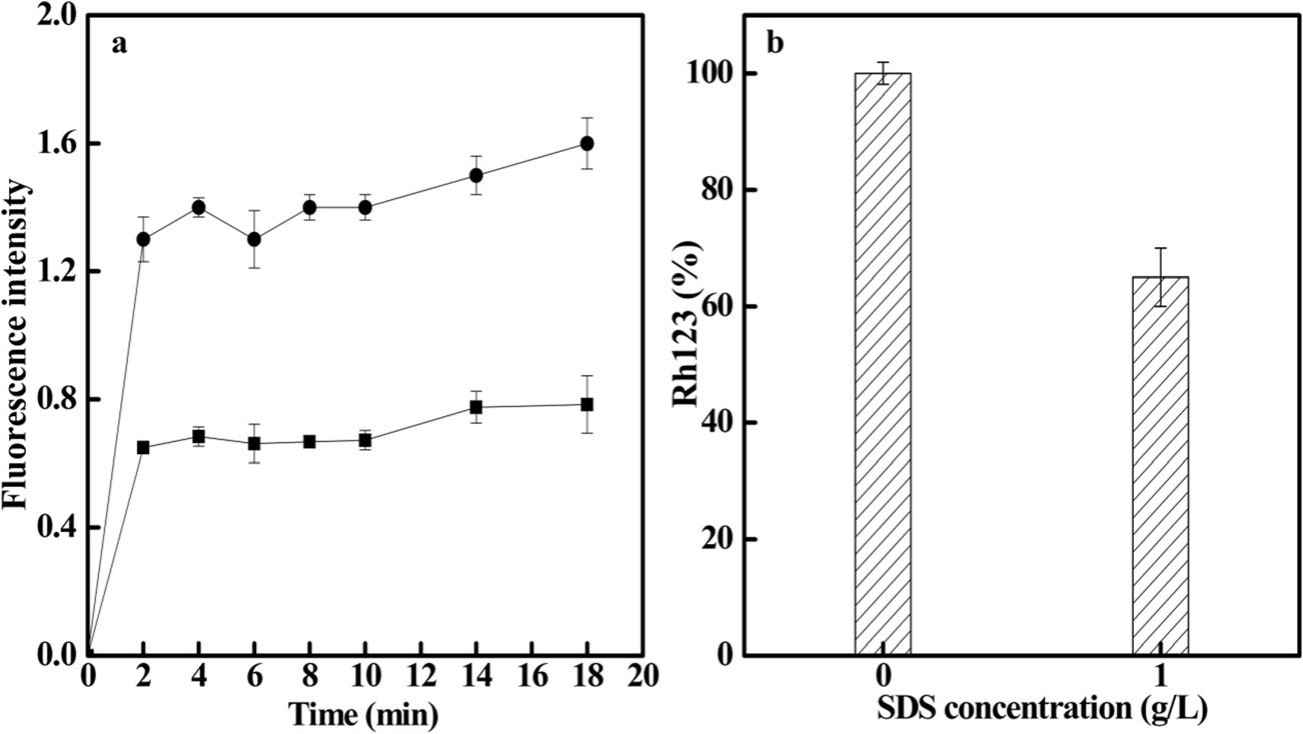
**a**, **b** The effects of SDS on NPN and Rh123 uptake (black square, 0 g/L, black circle, 1.0 g/L)

The OM can be monitored via the fluorescence increase due to N-phenyl-1-naphthylamine (NPN) partitioning into the hydrophobic core of the lipid bilayer, which occurs in a dose-dependent manner (RIbrahim et al. 2000). When SDS was mixed with *G. lozoyensis* cell suspensions, the NPN uptake was rapidly increased and reached its maximum at about 2 min and remained unchanged thereafter (Fig. 2a). Due to the dose-dependence of the partitioning, higher fluorescence intensity indicates a higher permeability of the cell membrane, which proved that the surfactant can improve the permeability of the cell membrane. Moreover, mitochondrial activity was also found to be affected by SDS. Rhodamine 123 (Rh123) directly and selectively stains mitochondria of living cells and is therefore used as a mitochondrial probe. As shown in Fig. 2b, the Rh123 (%) of the test group (1.0 g/L SDS) was about 65% of the value measured in the control group (0 g/L SDS). These results indicate that mitochondria are damaged by the addition of SDS.

### Effect of SDS on the morphology of *G. lozoyensis* during extractive fermentation

**Fig. 3:**
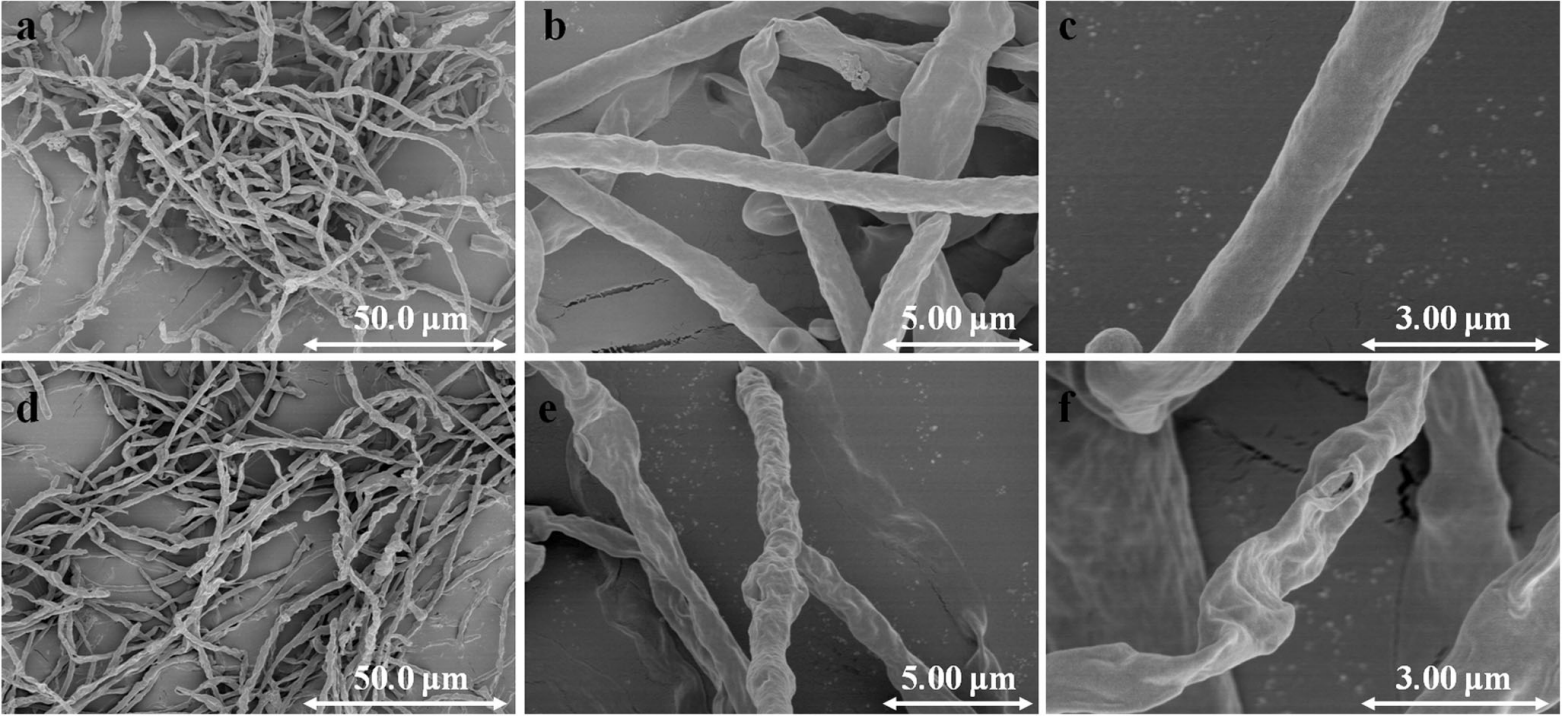
SEM images showing the morphology of hyphae from normal batch fermentation (**a**, × 1000; **b**, × 7000; and **c**, × 15,000) and extractive batch fermentation with 1.0 g/L SDS (**d**, × 1000; **e**, × 7000; and **f** × 15,000)

Filamentous fungi are morphologically complex microorganisms and a certain morphology is preferred to ensure maximal biological performance (Papagianni 2004). The addition of SDS had obvious effects on the morphology of the mycelia of *G. lozoyensis*, as it inhibited the development of both hyphae and pellets in extractive batch fermentation (Fig. 3 and Table 1). In normal batch fermentation, the *G. lozoyensis* hyphae grew well and the morphology was normal (Fig. 3a–c), with smooth and full single mycelia. The diameters of the hyphae and pellets were about 0.86 μm and 0.53 mm, respectively, and the DCW was 167.28 g/L. In the extractive batch fermentation, the morphological characteristics of *G. lozoyensis* were as shown in Fig. 3d–f. The surface of the single hyphae exhibited dense shrinkage and began to be uneven thickness. The hyphae and pellet diameters were about 0.74 μm and 0.49 mm, respectively, and the DCW was 200.40 g/L.

## Discussion

The extent of enhancement is closely associated with the type of extractant and its interaction with the microbial cells (Kang et al. 2013; Wang et al. 2013). Considering the benefit of using SDS as measured in the preliminary trials, we chose SDS as the addictive to explore the mechanism of extractive fermentation. After optimization, the addition of 1.0 g/L SDS on the 13th day of the fermentation process showed the best effect on pneumocandin yields. SDS is an amphiphilic compound that has both water and oil solubility, and its structure is similar to the structure of phospholipids molecules in the cell membrane. Consequently, the added SDS could form a complex with membrane phospholipids to form mixed micelles which would greatly alter the structure of the cell membrane and improve its permeability, making the membranes more conducive to the export of intracellular pneumocandin B_0_ (Le et al. 2000; Wei et al. 2003).

As shown in Table 1, in batch fermentation, 12.44% pneumocandin B_0_ was released into broth and others were accumulated in mycelium, which proved that pneumocandin B_0_ is an intracellular product. The change of pneumocandin B_0_ in supernatant (228.67 vs. 580.33, mg/L), increased by 153.78%, demonstrated that the addition of SDS accelerated the trans-membrane transport of intracellular pneumocandin B_0_ to the extracellular medium. On the other hand, the increase of ρ_2_ from 10.98 mg/g to 12.62 mg/g proved that the improvement of pneumocandin B_0_ yield was caused by the improved synthesis capacity of *G. lozoyensis*, caused by the release of intracellular pneumocandin B_0_. Based on the date of ρ_1_ (9.61 vs. 9.72 mg/g), we inferred that the synthesis of intracellular pneumocandin B_0_, in batch fermentation and extractive batch fermentation has reached its intracellular saturation level.

The fatty acid composition of the cell membrane has a great influence on permeability. The saturated fatty acids in the cell membranes are linear, with tight inter chain arrangements and large interactions, resulting in low penetrability of the membrane. Unsaturated fatty acids are bent, making it difficult for the two fatty acid chains of phospholipids to align close to each other, resulting in increased penetrability of the membrane (Robert et al. 2002). The increased unsaturated/saturated fatty acid ratio observed in this study implied that the fatty acid components in the cell membrane had changed upon the addition of SDS (Fig. 2a). With higher SDS concentration, a higher fluorescence intensity was obtained, which was in accordance with the reduced membrane integrity. Moreover, shrinkage of the surface of *G. lozoyensis* was observed in the extractive batch fermentation (Fig. 3e). Generally speaking, surfactants improve the permeability of the cell membrane, facilitate the release of intracellular secondary metabolites into the culture supernatant, alleviate product feedback inhibition, and enhance production accordingly (Kleinegris et al. 2011; Wang et al. 2013; Wei et al. 2003).

The addition of extractants, such as Tween-80, DMSO, and CTAB, were found to increase cell membrane penetrability and cause cytoplasm leakage, with lower cell viability or cell death (Brodelius and Nilsson 1983; Chen et al. 2007; Xing et al. 2009). Similarly, a 35% decrease of Rh123 (%) and the breakage of hyphae were observed in the extractive batch fermentation (Figs. 2b and 3e), which indicated that SDS in extractive batch fermentation destroys the cell membrane and reduces the cells’ viability. We speculated that this may be due to changes in cell membrane permeability that cause osmotic pressure changes, resulting in the loss of intracellular material and partial inactivation of mitochondria (Tao et al. 2011). Although the decrease of the diameters of hyphae and pellets (from 0.86 to 0.74 μm, 0.53 to 0.49 mm) indicated that SDS reduced the viability of *G. lozoyensis* cell, the DCW still increased when compared with no SDS addition. In our previous study (Song et al. 2018), we found that by controlling pellet diameter to be 0.3~0.5 mm, the dissolved oxygen during fermentation maintained above 30%, and the pneumocandin B_0_ yield and DCW increased by 40 and 18.8%, respectively. Similar behaviors were reported in other studies and the reason might be that smaller pellets were more conducive to sorption of dissolved oxygen and nutrients in extractive fermentation compared with the larger pellets (Metz and Kossen 1977; Nanou and Roukas 2010).

**Fig. 4:**
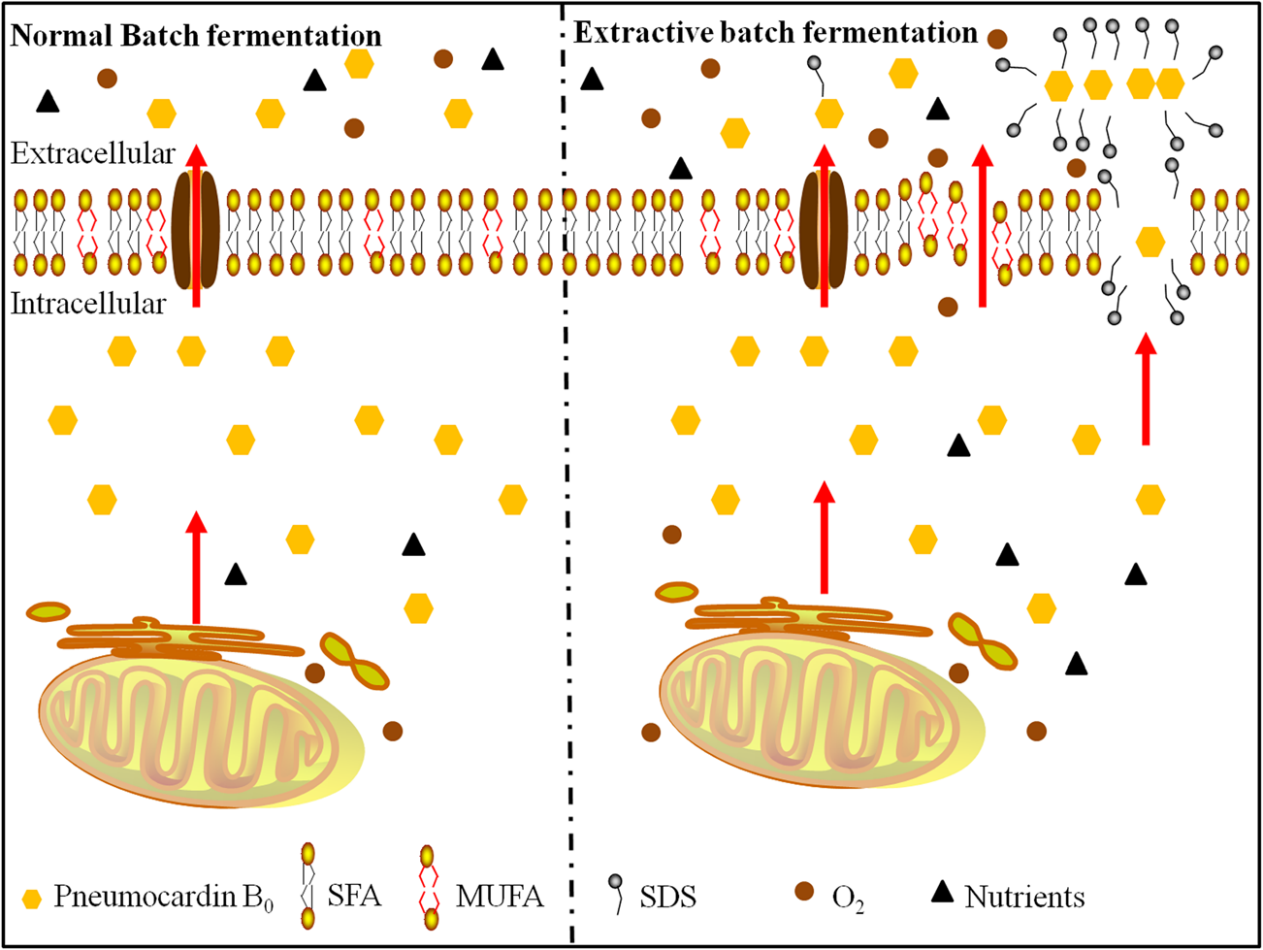
Trans-membrane secretion model of pneumocandin B_0_ in extractive batch fermentation

Based on the results, we postulated a putative trans-membrane release model of pneumocandin B_0_ in extractive fermentation (Fig. 4). With the addition of SDS, the increased incorporation of unsaturated fatty acids in the cell membrane and mixed micelles improved the membrane permeability, facilitating the release of intracellular pneumocandin B_0_ and allowing the intracellular synthesis of new pneumocandin B_0_. Furthermore, the reduction of hyphal and pellet diameters facilitates higher dissolved oxygen and more efficient exchange of nutrients to cells of *G. lozoyensis*.
